# Facilitators and barriers to improved cookstove adoption: a community-based cross-sectional study in Northwest Ethiopia

**DOI:** 10.1186/s12199-020-00851-y

**Published:** 2020-05-15

**Authors:** Mesafint Molla Adane, Getu Degu Alene, Seid Tiku Mereta, Kristina Lutomya Wanyonyi

**Affiliations:** 1 Department of Environmental Health, School of Public Health, College of Medicine & Health Sciences, Bahir Dar University, Bahir Dar, Ethiopia; 2 Department of Epidemiology and Biostatistics, School of Public Health, College of Medicine & Health Sciences, Bahir Dar University, Bahir Dar, Ethiopia; 3 Departments of Environmental Health Science and Technology, Jimma University, Jimma, Ethiopia; 4 Queen Mary University of London, Institute of Dentistry, Barts and The London School of Medicine and Dentistry, London, UK

**Keywords:** Adoption, Fuel, Household, Improved cookstove

## Abstract

**Background:**

Among the environmental risk factors, household air pollution exposure from traditional cooking practices is one of the biggest killers globally, which mainly impacts developing countries where many families rely on traditional cooking practices. Although improved cookstove adoption is central to tackle this public health issue, the efforts to disseminate cookstove technologies have faced challenges, and the adoption rates are reported to be very low in many developing countries including Ethiopia. Therefore, this study aimed to determine the magnitude and identify potential factors that may act as facilitators or barriers to adoption from users’ point of view.

**Methods:**

As part of the wider stove trial project, a cross-sectional study was conducted among a total of 5830 households under randomly selected clusters. The required data were collected through face-to-face interviews, and a backward stepwise logistic regression analysis technique was applied to evaluate the effect of potential predictor variables on adoption using adjusted odds ratio (AOR) as measures of effect.

**Results:**

The prevalence of adoption was found to be 12.3% (95% CI 11.5–13.2), and households headed by females (AOR 1.96; 95% CI 1.24–3.10), private house ownership (AOR 4.58; 95% CI 3.89–6.19), separate cooking location (AOR 1.84; 95% CI 1.49–2.78), fuel purchasing (AOR 2.13; 95% CI 1.64–2.76), health benefit (AOR 1.76; 95% CI 1.15–2.70), optimistic social interaction (AOR 1.81; 95% CI 1.46–2.26), traditional suitability (AOR 1.58; 95% CI 1.28–1.95), stove use demonstration experience (AOR 2.47; 95% CI 1.98–3.07), cheap price (AOR 2.48; 95% CI 1.91–3.21), availability (AOR 1.81; 95% CI 1.5–1, 2.17), fuel-saving benefit (AOR 1.63; 95% CI 1.18–2.24), and more durable stove (AOR 1.71; 95% CI 1.30–2.26) of cookstove played a significant role as facilitators to adoption. In addition, lower educational level of head (AOR 0.31; 95% CI 0.23–0.42) and fuel processing requirement (AOR 0.55; 95% CI 0.44–0.70) of cookstove were found to be barriers for adoption.

****Conclusions**:**

Extremely lower improved cookstove adoption was observed due to household- and setting-related, cookstove technology-related, user knowledge- and perception-related, and financial- and market development-related factors. Therefore, to gain successful adoption, implementers and policymakers should consider those important factors in the implementation of clean cooking solutions to the community.

## Background

Among environmental risk factors, household air pollution (HAP) exposure from traditional cooking practices is one of the biggest killers globally [1]. Nearly 600,000 Africans die annually and millions more suffer from illnesses caused by HAP [2]. Improved cookstove (ICS) adoption is central to tackling this public health issue, which mainly impacts on developing countries where many families rely on traditional cooking practices [3]. ICS adoption has the potential to generate a variety of health, social, economic, and environmental benefits in sub-Saharan Africa countries [4, 5] including Ethiopia [6–9]. It is the most affordable [10] and sustainable intervention [11] when compared to other technologies. The adoption of ICS would also contribute towards meeting at least five of the Sustainable Development Goals (SDGs), particularly SDG 7, which seeks to ensure affordable, reliable, sustainable, and modern energy for all by 2030 [12, 13].

Currently, it would appear that global ICS distribution programs are quickly expanding and being implemented around the world [14]. For example, the Global Alliance for Clean Cookstoves has set a goal to reach 100 million homes with cleaner and more efficient cooking methods by 2020 [13]. Nevertheless, evidence suggests that despite the noticeable benefits and the accompanying interventions, the efforts to disseminate various types of ICS technologies have faced challenges in adoption in most sub-Saharan countries [15–18]. In Ethiopia, ICS initiatives have been started with the introduction of electric *injera* baking stoves and wood saving biomass cookstoves in the early 1970s, and large-scale distribution to consumers was started in the mid-1980s with the introduction of the *Lakech* charcoal stove followed by the *Mirt injera* baking biomass stove and the *Tikikil* wood stove and then by the national biogas program which have been promoted since 1998, 2009, and 2009 respectively [19]. Although the history of ICS initiatives goes as far back as the early1970s, the adoption rate is reported to be very low [20, 21]. Only 10% (fewer than 6% in rural) of Ethiopian households have adopted ICS as evidenced by recent reports [19, 22]. As a result, about 94% of the Ethiopian population remains without access to clean cooking services, making it the highest compared with average global and sub-Saharan Africa proportions which are 38% and 84% respectively [4].

The factors that limit or enable people’s decision-making around ICS adoption are thought to be wide-ranging and contextual [9, 20, 23], and the debate on adoption drivers is still extremely open in the literature [24]. In Ethiopia, a recent systematic review of the existing literature identified some barriers to the adoption of clean household energy which are broad and mostly lie with the challenges in disseminating ICS technology from the provider’s responsibility side. These are lack of coordination, un-affordability, unclear regulatory responsibility, lack of awareness, insufficient market development, cultural factors, and inadequate electricity supply [23].

However, ICS adoption studies are scarce and scattered from the users’ point of view of compatibility to their current situation [25], and identifying the factors that may act as facilitators or barriers to adoption [26, 27] and blending them into implementation programs [28] and policies [29] are critical to ensure optimum adoption [30, 31]. Furthermore, since most adoption studies in Ethiopia focus on a particular cookstove type, there is limited information on the general attributes of cookstoves in Ethiopia, and this information is vital for designing and implementing an effective cookstove program.

Therefore, the main goal of the present study was to determine the magnitude and identify factors that may act as facilitators or barriers to ICS adoption at the household level from users’ point of view of compatibility to their current situation in Northwest Ethiopia.

## Methods

### Study design and setting

As part of the wider stove trial project (ClinicalTrials.gov Identifier: NCT03612362), a community-based cross-sectional study was employed in May 2018. This study had been conducted in a low-income rural community of the Mecha Health and Demographic Surveillance System (MHDSS) site in which biomass fuel use is a major household energy source for cooking. MHDSS site is a field research center established in 2013 by Bahir Dar University to conduct and support postgraduate-level studies in the region. It is located 525 km away from the capital city of Ethiopia, Addis Ababa, towards Northwest and 40 km far away from the capital city of Amhara Regional State, Bahir Dar. The study area comprises three major climatic zones of highlands (*Dega*), midlands (*Weina Dega*), and lowlands (*Kola*). It also consists of 10 randomly selected *Kebeles* (sub-districts/the smallest administrative unit) of three urban and 7 rural with a total of 132 clusters/*Gots*. According to the official population profile report of MHDSS, a total of 20,631 households were registered at the end of 2016.

### Sample size determination

The sample size of this study was calculated using the population survey formula on EPI INFO program, assuming a 95% confidence level, a 3% acceptable margin of error, a 50% acceptable estimated population proportion of ICS adoption, and a design effect of 2 for cluster randomization technique and allowing a 5% oversampling to account for any unpredictable events in the field. The final estimated sample size was 2245 households. However, since we had the advantage of using all the baseline data of the wider stove trial project (ClinicalTrials.gov Identifier: NCT03612362), we considered all the randomly selected 100 clusters containing about 5830 eligible children from the stove trial project in order to attain the advantage of having a larger sample size which would reduce the sampling error, thereby increasing the accuracy of estimations.

### Sampling and recruitment of participants

Cluster randomization technique was used to select the required study participants, and the cluster units were the small villages termed as *Gote* in the local language. Among the total 132 clusters in the MHDSS site, 100 clusters were selected randomly to represent the total population. Households with any type of cooking practice were eligible for the study in all clusters, and all (5830) eligible households within the selected clusters were included in the study. The selected households were identified using the permanent MHDSS site house number, and the actual data collection was carried out from May 1 to 31 in 2018.

### Variables, data sources, and methods of assessment

ICS adoption refers to the use of any ICS as a major source of energy, such as improved solid fuel, liquefied petroleum gas, biogas, solar cookers, alcohol (ethanol and methanol), and electrical energy stoves [32]. ICS adoption status was measured as a binary variable carrying a value of “Yes” for adopters and “No” for non-adopters. The predictor variables were grouped into four major categories as household- and setting-related, cookstove technology-related, users’ knowledge- and perception-related, and financial- and market development-related factors. In this study, wide-ranging potential predictor variables of adoption were assessed through face-to-face interviews followed by an observational checkup in the selected households as illustrated in detail next:

1. Household- and setting-related characteristics

Under this category, socio-demographic-, household-, and setting-related potential cookstove adoption factors were investigated in this study as briefly discussed next:

- Gender of the household head: Refers to the role of household head gender in stove adoption, measured as female- or male-headed household.
- Educational status of the household head: Refers to the role of an educational level attained by the household head in stove adoption. It was assessed by classifying into five categories as (i) do not read and write, (ii) read and write only, (iii) primary schooling completed, (iv) secondary schooling completed, and (v) tertiary schooling completed.
- Family size of the household: Refers to the role of the total number of individuals permanently living in the household in ICS technology adoption, and it was assessed by classifying into four categories as (i) 2–3 individuals, (ii) 4–5 individuals, (iii) 6–7 individuals, and (iv) 8 or more individuals.
- Number of rooms: Refers to the role of adequate space inside the main living house for placing a permanent stove in ICS technology adoption as measured by the total number of rooms in the main living house, and it was assessed by classifying into four categories as (i) one room, (ii) two rooms, (iii) three rooms, and (iv) four or more rooms.
- House ownership: Refers to the role of house ownership status in stove adoption, measured as private/own or rented.
- Location of cooking quarter: Refers to the role of a cooking quarter location in stove adoption as assessed through observing and asking respondents about the location of the main cooking quarter of the household by classifying into (i) separate kitchen and (ii) inside the living house.
- Fuel source: This refers to the role of a fuel source as assessed by asking respondents about their main source of fuel for household cooking purposes by classifying into three categories as (i) purchasing, (ii) purchasing and collecting, and (iii) collecting.
- Multiple stove use: Refers to the multiple stove use or “stove stacking” status of the households as assessed through observing and asking respondents whether the household currently used more than one stove technology in parallel to meet cooking needs of the household or not as measured by classifying into three categories as (i) yes, (ii) no, and (iii) not applicable.

2. Cookstove technology-related factors

This category refers to the link of perceived features of cookstove technology with stove adoption at the household level, and six technology-linked factors were investigated as briefly pointed out next:

- Fuel processing: This refers to the importance of prior fuel processing requirement of a stove in adoption as assessed through asking respondents whether the current cookstove of the household requires prior fuel processing to prepare local dishes or not (yes/no).
- ICS stove durability: This refers to the role of perceived stove durability in adoption as assessed by asking respondents about the durability of ICS technologies compared to the traditional cookstove types as measured by classifying into three categories: (i) more durable, (ii) comparable, and (iii) less durable.
- Fuel-saving benefit: This refers to the role of perceived importance of the fuel-saving benefit of ICS technology in stove adoption as assessed through asking respondents about the importance of fuel-saving characteristic of ICS technology compared to the traditional cookstove type and measured by classifying into three categories as (i) important, (ii) neutral, and (iii) less important.
- Health benefit: This refers to the importance of a perceived health benefit of ICS technology in adoption. In the present investigation, the respondents were asked to give a response about the importance of ICS technology in reducing wood smoke exposure and health risk such as eye/throat irritation or respiratory diseases compared to the traditional cookstove type and measured by classifying into three categories as (i) important, (ii) neutral, and (iii) less important.
- Time-saving benefit: This refers to the importance of perceived cooking time-saving benefit of a stove technology in adoption, and it was assessed through asking respondents about the value of the time-saving characteristic of ICS technology in adoption compared to the traditional cookstove type and measured by classifying into three categories as (i) important, (ii) neutral, and (iii) less important.
- Safety benefit: This refers to the value of the perceived importance of safety benefits in ICS technology adoption. In this study, the key safety concern was child burn injury prevention capacity as assessed by asking respondents about the importance of safety benefits from using ICS technology compared to the traditional cookstove type and measured by classifying into three categories as (i) important, (ii) neutral, and (iii) less important.

3. Cookstove users’ knowledge- and perception-related factors

This category deals with the role of users’ knowledge- and perception-related factors in stove adoption at the household level, and the following factors were investigated under this category as pointed out briefly next:

- Social interaction: This refers to the role of social interaction in cookstove technology adoption as assessed through asking respondents whether they had been previously convinced by someone such as neighbors and relatives who had adopted ICS technology to adopt ICS technology for the household or not (yes/no).
- Traditional suitability of cookstove: This refers to the role of traditional suitability of cookstove in adoption as assessed by asking respondents whether they believed that currently distributed ICS technologies are suitable for preparing the usual traditional meals of the household or not (yes/no).
- Demonstration experience on stove use: Refers to the role of stove promotion strategy in ICS technology adoption. It was assessed through asking respondents about the availability of ICS technology promotion strategy within the locality as measured by their previous experience of live ICS use demonstration by promoters about the use of any new ICS technology in order to purchase for the household or not (yes/no).

4. Financial- and market development-related characteristics

This category refers to the role of financial approaches and market development strategies in stove adoption at the household level, and the next characteristics were considered under this category as follows:

- Stove price: This refers to the role of a perceived price of ICSs in adoption that may influence users to maintain or switch their cookstove technology. It was assessed through asking the head of the household about the overall cost of the local ICS technologies by classifying into four categories as (i) cheap, (ii) medium, (iii) expensive, and (iv) do not know.
- ICS availability: This refers to the role of ICS availability in adoption as assessed by asking respondents regarding the availability of ICS technologies (yes/no).

### Data collection

The required data were collected from May 1 to 31 in 2018 by trained professionals through face-to-face interviews using a structured questionnaire and direct verification (observation) whenever essential to capture ICS adoption-linked data. Several special efforts were applied before and during the data collection period to prevent missing data and avoid the associated complexities in data analysis and interpretation. To mention some, the stove trial research project was publicized through a live discussion on the Regional Television show in May 2018 to build community interest in the study. Interest in the study was also created through communications about the study during the regular local health development army team meetings as well as through home visits by the local health and agricultural extension workers along with local energy experts by means of working on active community engagement through the Ethiopian health and agricultural extension programs as well as through the local health development army team structure.

Participants were also informed that they will receive an improved baking stove for free either at the initiation or at the end of the study to maintain justice and to help achieve a high level of research participation. Besides, we utilized local household energy experts and health extension workers to oversee the overall efforts in recruiting eligible households. Home visits for data collection were scheduled and communicated. Repeated home visits were made to deal with missed data using the permanent MHDSS site house number to identify missed households as well as data collectors training with practical exercises on how to avoid non-response and application of data collection manual were the major efforts to achieve complete participant enrolment.

### Data quality assurance

Prior to the actual data collection, emphasis was given in designing the study tool and training was given for data collectors with a practical exercise on how to collect the required data as well as the study tool was pretested in the nearby district, which was not part of the study area, and the necessary amendments were done on the flow and clarity of some questions to suit respondents. During the actual data collection, a data collection manual was used by each data collector to facilitate the data collection process and about 5% of the already surveyed households were randomly selected and re-interviewing took place at the time of data collection as cross-checking mechanism to ensure the validity of the collected data.

### Statistical methods

Backward stepwise logistic regression analysis technique was applied to evaluate the effect of potential predictor variables on ICS adoption using AORs as a measure of effect with the respective 95% confidence intervals (CIs). All statistical tests were two-sided with *p* value < 0.05 considered statistically significant. Hosmer and Lemeshow test of the goodness of fit was checked, and the model was a good fit for the data with a *p* value of 0.406. Besides model fitness, multicollinearity diagnosis was also carried out using the correlation matrix method, and all values were well below 0.20, which confirmed that multicollinearity among the predictor variables was not a concern for the model.

Descriptive statistical analyses were done to describe the characteristics of the outcome and the independent variables, and univariate logistic regression model analyses that are the association of ICS adoption with each predictor variable independently which provide crude odds ratios (CORs) for the associations were carried out to describe the independent association of ICS adoption with each predictor variable. Multivariable logistic regression model analysis (i.e., the association of ICS adoption with multiple independent variables simultaneously which provide AORs for the associations) was conducted to describe the associations between ICS adoption and all independent variables by adjusting for the effect of each predictor variable at the same time [33]. Accordingly, both crude and adjusted odds ratios were obtained by taking the exponentials of the beta coefficients, labeled as Exp(*ß*) (i.e., Exp(*ß*) = odds ratio) from the univariate and multivariable logistic regression analysis outputs respectively using the Statistical Package for Social Sciences (SPSS) for Windows version 22. Then, the results describing the association between the dependent variable (ICS adoption) and the independent variables are presented in CORs and AORs with the respective 95% CIs. At last, the paper is reported following the STROBE (Strengthening the Reporting of Observational Studies in Epidemiology) statement to address the essential components of the report [34].

## Results

### Socio-demographic characteristics of study participants

A total of 5830 eligible households were included in the study. Out of which, most (92.7%) households were male-headed and a significant number (61.5%) of heads were unable to read and write. About 63% of the households used a separate kitchen, and most (89.1%) houses were owned privately as shown in Table 1.

**Table 1:** Household and setting characteristics of study participants in Northwest Ethiopia, 2018 (*n* = 5830)

Characteristics		Number	Percent
Gender of the household head	Female	424	7.3
	Male	5406	92.7
Educational status of the household head	Do not read/write	3583	61.4
	Read/write only	843	14.5
	Primary school	624	10.7
	Secondary school	385	6.6
	Higher education	395	6.8
Family size of the household	2–3	1158	19.9
	4–5	2025	34.7
	6–7	1784	30.6
	8 or more individuals	863	14.8
Number of rooms in the main living house	One room	1384	23.8
	Two rooms	3203	54.9
	Three rooms	1101	18.9
	Four or more rooms	142	2.4
House ownership status	Private/own	5194	89.1
	Rented	636	10.9
Location of cooking quarter of household	Separate kitchen	3685	63.2
	Inside living house	2145	36.8

3.1. Improved cookstove technology adoption

The prevalence of ICS technology adoption was 12.3% (95% CI 11.5–13.2) in the study area, and the majority (62.2%) of the adopter households had used their ICS for more than 2 years from acquisition. In this study, multiple stove use (stove stacking) was a common practice with about 64% of the adopter households currently used more than one stove technology in parallel to meet their household cooking needs as indicated in Table 2.

**Table 2:** Improved cookstove adoption status of study households in Northwest Ethiopia, 2018

Characteristics		Number	Percent
ICS adoption status of households (*n* = 5830)	No	5110	87.7
	Yes	720	12.3
Duration of ICS adoption (*n* = 720)	Acquisition (ICS installed)	19	2.7
	Initial adoption (used < 1 year)	114	15.8
	Medium-term adoption (used 1–2 years)	139	19.3
	Long-term adoption (ICS used > 2 years)	448	62.2
Multiple cookstove use among adopters (*n* = 720)	Yes	459	63.7
	No	261	36.3

### Facilitators and barriers to improved cookstove

The influence of various potential factors, which may act as facilitators or barriers to ICS adoption at the household level, was estimated by fitting both crude and adjusted binary logistic regression models. As summarized in Table 3, our findings suggest that households headed by females (AOR 1.96; 95% CI 1.24–3.10), private house ownership (AOR 4.58; 95% CI 3.89–6.19), separate cooking location (AOR 1.84; 95% CI 1.49–2.78), fuel purchasing (AOR 2.13; 95% CI 1.64–2.76), positive perceived health benefit of cookstove (AOR 1.76; 95% CI 1.15–2.70), optimistic previous social interaction (AOR 1.81; 95% CI 1.46–2.26), traditionally suitable (AOR 1.58; 95% CI 1.28–1.95), previous live demonstration experience on ICS use (AOR 2.47; 95% CI 1.98–3.07), perceived cheap price of cookstove (AOR 2.48; 95% CI 1.91–3.21), availability of cookstove (AOR 1.81; 95% CI 1.5–1, 2.17), positive perceived fuel-saving benefit (AOR 1.63; 95% CI 1.18–2.24), and perceived more durability of cookstove technology (AOR 1.71; 95% CI 1.30–2.26) played a significant role as facilitators to adoption within the investigated households.

**Table 3:** Logistic regression analyses describing the associations between ICS adoption and possible predictor variables in Northwest Ethiopia, May 2018 (*n* = 5830)

Characteristics		ICS adoption		COR^a^ (95% CI)	AOR^b^ (95% CI)
		No	Yes		
Gender of the household head	Female	287	137	3.95 (3.17, 4.93)	1.96 (1.24, 3.10)*
	Male	4823	583	1	
Educational level of the household head	Do not read/write	3319	264	0.14 (0.11, 0.17)	0.31 (0.23, 0.42)*
	Read/write only	758	85	0.19 (0.14, 0.26)	0.41 (0.29, 0.58)*
	Primary school	513	111	0.37 (0.28, 0.50)	0.50 (0.35, 0.69)*
	Secondary school	270	115	0.73 (0.54, 0.99)	0.94 (0.67, 0.1.33)
	Higher education	250	145	1	
Family size of the household	2–3	972	186	1.80 (1.37, 2.37)	0.73 (0.53, 1.01)
	4–5	1745	280	1.51 (1.16, 1.95)	0.95 (0.70, 1.27)
	6–7	1613	171	1.00 (0.76, 1.31)	0.93 (0.68, 1.26 )
	8 or more individuals	780	83	1	
House ownership	Private/own	4544	650	1.16 (0.89, 1.50)	4.58 (3.89, 6.19)*
	Rented	566	70	1	
Location of cooking quarter	Separate kitchen	3106	579	2.65 (2.19, 3.21)	1.84 (1.49, 2.78)*
	Inside living house	2004	141	1	
Source of fuel	Purchasing	1070	346	4.23 (3.48, 5.14)	2.13 (1.64, 2.76)*
	Purchasing and collecting	1727	197	1.49 (1.21, 1.84)	1.37 (1.08, 1.75)*
	Collecting	2313	177	1	
Fuel processing requirement	Yes	1379	120	0.54 (0.44, 0.67)	0.55 (0.44, 0.70)*
	No	3731	600	1	
Durability of cookstove	More durable	2203	448	2.19 (1.71, 2.80)	1.71(1.30, 2.26)*
	Comparable	2004	188	1.01 (0.77, 1.32)	1.22 (0.91, 1.64)
	Less durable	903	84	1	
Fuel-saving benefit of cookstove	Important	2903	546	2.70 (2.01, 3.62)	1.63 (1.18, 2.24)*
	Neutral	1447	121	1.20 (0.86, 1.68)	1.38 (0.96, 1.98)
	Less important	760	53	1	
Health benefit of cookstove	Important	378	180	4.43 (3.52, 5.57)	1.76 (1.15, 2.70)*
	Neutral	2826	335	1.10 (0.92, 1.32)	0.98 (0.80, 1.19)
	Less important	1906	205	1	
Time-saving benefit of cookstove	Important	1842	376	2.24 (1.80, 2.79)	1.19 (0.93, 1.54)
	Neutral	1983	227	1.26 (1.00, 1.59)	0.89 (0.69, 1.16)
	Less important	1285	117	1	
Safety benefit of ICS	Important	2320	348	1.03 (0.81, 1.29)	1.13 (0.87, 1.48)
	Neutral	2058	265	0.88 (0.69, 1.12)	1.06 (0.81, 1.38)
	Less important	732	107	1	
Optimistic previous social interaction	Yes	3349	585	2.28 (1.87, 2.77)	1.81 (1.46, 2.26)*
	No	1761	135	1	
Traditional suitability of cookstove	Yes	760	189	2.04 (1.70, 2.45)	1.58 (1.28, 1.95)*
	No	4350	531	1	
Live demonstration experience	Yes	3024	592	3.19 (2.62, 3.89)	2.47 (1.98, 3.07)*
	No	2086	128	1	
Price of cookstove	Cheap	461	142	2.50 (2.01, 3.13)	2.48 (1.91, 3.21)*
	Medium	2161	272	1.02 (0.86, 1.22)	1.00 (0.82, 1.21)
	Expensive	2488	306	1	
Availability of cookstove	Yes	2226	468	2.41 (2.05, 2.83)	1.81 (1.51, 2.17)*
	No	2884	252	1	

*Significantly associated

COR^a^ and AOR^b^ were obtained by taking the exponentials of the beta coefficients (i.e., Exp(*ß*) = odds ratio) together with their corresponding 95% CIs from our univariate and multivariable logistic regression model analysis outputs respectively using SPSS for Windows version 22 to describe the associations between ICS adoption and the possible predictor variables. The AORs (95% CIs) were obtained from one logistic regression analysis model including an outcome variable of ICS adoption and all independent variables simultaneously

In addition, the lower educational level of the household head (AOR 0.31; 95% CI 0.23–0.42) and fuel processing requirement of cookstove technology (AOR 0.55; 95% CI 0.44–0.70) were found to be barriers for adoption in the study community.

## Discussion

This study investigated the magnitude and factors associated with cookstove technology adoption among a total of 5830 households. The prevalence of ICS technology adoption was 12.3% (95% CI 11.5–13.2) in the study households. This finding was extremely lower in comparison with the finding of an adoption study conducted in Southeastern Ethiopia, which reported that the majority of the respondents (75%) have adopted ICS in 2015 [35]. The possible reasons for this big disparity might be the Southwestern Ethiopia study was conducted in an urban setting, and the study area was purposively selected as the study site due to better accessibility of different types of ICS cookstoves to the inhabitants [35]. The other possible explanation for the difference in adoption might be the reflection of the difference in geographical location and cultural variations. In general, though ICS adoption is believed to generate a variety of public health and environmental benefits in sub-Saharan Africa countries [4, 5] including Ethiopia [6–9], this study revealed a very low adoption which might expose all household family members to high HAP and its health outcomes. Hence, this finding may encourage consumers, implementers, and policymakers to play a role in ICS adoption efforts.

Pertaining to the factors of adoption, we investigated 17 potential factors that may act as facilitators or barriers to ICS adoption at the household level in the study area as shown in Table 3 and a narrative description of the major findings is discussed in four major categories below.

### Household- and setting-related factors

Under this category, gender, education, house ownership, cooking quarter location, and fuel source type were found to be significantly associated with adoption in the study area as discussed next. To begin with gender, the odds of ICS adoption in female-headed households were nearly twice more than male-headed households in the study population (AOR 1.96; 95% CI 1.24–3.10). This might be due to the fact that the responsibility of cooking-related activity is traditionally given to women in Ethiopia, and it may have driven women who participated in the cooking and made decisions as household heads to adopt ICS due to their first-hand experience. The finding of this study is in conformity with the finding of an adoption study, which reported that households headed by a male were less likely to adopt ICS than those headed by a female in southern Ethiopia (OR 0.251, 95% CI 0.068–0.916) [20]. However, this was in contrast with the finding of an adoption study conducted in Southwest Ethiopia, which reported that the gender of the household head was not significantly associated with adoption [9].

Concerning the role of education, households headed by those who could not read and write were 69% less likely to adopt ICS than households headed by those who have completed higher education (AOR 0.31; 95% CI 0.23–0.42). The possible explanation for this finding might be that educated household heads were more informed about the benefits gained from the ICS adoption than uneducated household heads, and better education may increase awareness of the negative effects of the traditional cookstoves. This finding is similar to the finding of an adoption study conducted in South Ethiopia, which reported that educational status was associated with ICS adoption [20]. Nevertheless, the finding of this study is not in agreement with the finding of an adoption study conducted in Southwest Ethiopia, which reported that household head education was not significantly associated with adoption [9].

In this study, privately owned households were about four times more likely to adopt ICS than renting households (AOR 4.58; 95% CI 3.89–6.19). This might be explained by permanent home may favor ICS uptake in the study community as supported by previous studies which reported that homeownership and having a permanent house were reported to increase willingness to adopt ICS in Ethiopia [36], Mexico [37], and Bangladesh [38]. Besides homeownership, using a separate kitchen was also positively linked to ICS adoption in the study households compared with using the main living house as a main cooking quarter (AOR 1.84; 95% CI 1.49–2.78). The finding is supported by a previous study, which reported that the use of the kitchen as a bedroom was contributing factors to abandoning the use of ICS in rural Mexico [39].

The fuel source of the household was also found to be significantly associated with adoption. Respondents who reported fuel purchasing as their main source of fuel were about two times more likely to adopt ICS than respondents who reported a free fuel collection method (AOR 2.13; 95% CI 1.64–2.76). The possible reason for the present finding might be the high expenditure for fuel; households who purchase fuel might be positively influenced to adopt ICS due to the ongoing cost incurred to purchase fuel compared to those households who get fuel from free the collection method. This finding is in agreement with previous studies, which reported that households who purchase fuel were more likely to adopt ICS than those who could get free fuel [36], and the availability of free fuel was a factor leading to non-adoption of the ICS in Southeastern Ethiopia [35].

### Cookstove technology-related factors

Among the investigated technology-linked factors, the odds of ICS adoption among respondents who reported prior fuel processing requirements of ICS technology were 45% lower than those among respondents who do not report prior fuel processing requirements (AOR 0.55; 95% CI 0.44–0.70). This implies that ICS that does not require prior fuel processing might be more readily adopted by the community. The impact of the perceived importance of the fuel-saving benefit of ICS was also found to be positively associated with adoption (AOR 1.63; 95% CI 1.18–2.24). The possible explanation for this finding could be the greater value given to the fuel-saving benefit of ICS by the study community due to the high cost of fuel. This is supported by the previous stove adoption study, which found a positive association between the fuel-saving efficiency of ICS and adoption in Northern Ethiopia [6]. In addition, the importance of the perceived health benefit of ICS was also found to be positively associated with adoption (AOR 1.76; 95% CI 1.15–2.70). The finding is supported by a previous study, which reported that ICSs with smoke reduction feature were highly preferred by women in rural Senegal [40].

Furthermore, the perceived durability of ICS technology was also found to be positively associated with adoption (AOR 1.71; 95% CI 1.30–2.26). The likely explanation for the present finding might be that the expenditure for cookstove might negatively influence adoption due to the ongoing cost incurred to purchase ICS. This finding is in agreement with the previous study, which reported that durability as the main determinant factor for adoption in Ethiopia [9], ensuring cookstove durability facilitates adoption [27]. In general, all the cookstove technology-related findings of this study indicate that clean cooking technology program planners and implementers should think about the provision of a range of ICS technologies that have the potential to meet consumer needs while delivering significant health and environmental benefits.

### Cookstove users’ knowledge- and perception-related factors

Among the investigated knowledge- and perception-related factors, ICS adoption was significantly influenced by previous social interaction, and respondents who report an experience of optimistic previous social interaction with neighbors and relatives who had already adopted the ICS was found to increase the odds of ICS adoption (AOR 1.43; 95% CI 1.11–1.83) than respondents who do not report. Similarly, a previous study also reported that peer interaction outside of the household was a strong determinant factor of ICS adoption in urban Rwanda [41]. Our finding is also in agreement with a multi-country study conducted in Kenya and Zambia which reported that people are strongly influenced by their peers when making clean cookstove purchasing decisions [26].

Besides social interaction, traditionally, suitability of ICS was also found to be positively associated with adoption, respondents who reported that currently distributed ICSs are traditionally suitable were more likely to adopt than those who reported not suitable for preparing the usual meals of the household (AOR 1.58; 95% CI 1.28–1.95). This implies that an ICS that meets cooking expectations of the family for preparing the usual meals with the usual cooking habits could be more adopted due to the high flexibility of the device in meeting a wide range of operating conditions required by the local cooking practices of the households. The result was consistent with that of previous studies that reported the perceived potential of ICS to meet local needs for cooking a staple dish was a significant factor for adoption in rural Mexico [37], Kenya [42], and Northern Ghana [43].

Furthermore, the role of a live demonstration of ICS use in adoption was also investigated. Accordingly, previous experience of a live demonstration of ICS use was found to be positively associated with adoption (AOR 2.47; 95% CI 1.98–3.07). Likewise, live demonstration of ICS use to consumers encourages adoption as indicated by previous adoption study that asserted new ICS promotion to potential customers was a key factor in determining adoption in South Ethiopia with an odds ratio of 6.391 [20]. In general, the cookstove users’ knowledge- and perception-related findings of this study indicate that households have considerable values on some attributes of ICS technology such as traditional suitability, and adoption can be strongly influenced by local social interaction and previous live demonstration of ICS use. Hence, it is important to understand which aspects of ICS technology are most important to users, and cooking technology program implementers should take cookstove users’ knowledge and perceptions into consideration to increase adoption.

### Financial- and market development-related factors

In relation to financial implications of stove technology in adoption, households headed by those who reported that the price of new ICS is cheap were about two times more likely in the odds of ICS adoption than those households headed by those who perceived the price of new ICS is expensive (AOR 2.48; 95% CI 1.91–3.21). Similarly, it appears that stove price impacted the willingness to adopt ICS as shown by previous adoption studies in South [20] and urban Ethiopia [36]. The finding is also further supported by another previous study in Africa, which reported that technology cost was a significant barrier for adoption in Cameroon [44].

In addition, the role of market development in adoption was also investigated. Accordingly, cookstove availability was found to be positively associated with adoption with AOR of 1.81 (95% CI 1.51–2.17). The finding is supported by a previous study, which reported that the reason for most non-adopters (64.25%) in Ethiopia was the unavailability of ICSs [45], and another adoption study also reported that availability was an important factor for adoption in Cameroon [44]. Hence, these findings of financial- and market development-related factors imply that the price and availability of ICS technologies have a strong influence over adoption and are important factors to increase ICS adoption.

## Strengths and limitations

Our study has several strengths. It was community-based with a large sample size, strict application of data collection manual, and cross-checking mechanisms and considers a broad range of predictor variables. However, there may be important seasonal variations in cooking practices in the study population; thus, the point prevalence estimate, which might be influenced by season during which the survey took place was the main limitation of our study. Also, we considered only households with children from the wider cookstove trial project to attain the advantage of having a larger sample size which would reduce the sampling error thereby increasing the accuracy of estimations. On the other hand, selection bias might be introduced in doing so, and though we did everything possible to deal with it, still possible bias might have existed and considered as a limitation of the current study.

## Conclusions

In summary, a lower ICS adoption was observed due to household-related, cookstove technology-related, users’ knowledge- and perception-related, and financial- and market development-related factors. Thus, to gain successful ICS adoption, clean cooking technology planners, implementers, and policymakers should consider those most important factors in the design and implementation of ICS technology solutions in Ethiopia and other developing countries.

## Data Availability

The data that support the findings of this study are available from the corresponding author on reasonable request and with permission of the “Mecha” Health and Demographic Surveillance research center at Bahir Dar University in Ethiopia.

## Abbreviations

AOR: Adjusted odds ratio
CI: Confidence interval
COR: Crude odds ratio
HAP: Household air pollution
ICS: Improved cookstove
MHDSS: Mecha Health and Demographic Surveillance System
STROBE: Strengthening the Reporting of Observational Studies in Epidemiology
SPSS: Statistical Package for Social Sciences
